# *LsMybW*-encoding R2R3-MYB transcription factor is responsible for a shift from black to white in lettuce seed

**DOI:** 10.1007/s00299-023-03124-4

**Published:** 2024-01-11

**Authors:** Kousuke Seki, Kenji Komatsu, Kanami Yamaguchi, Yoshinori Murai, Keiji Nishida, Ryohei Koyama, Yuichi Uno

**Affiliations:** 1 Nagano Vegetable and Ornamental Crops Experiment Station, Tokoo 1066-1, Souga, Shiojiri, Nagano 399-6461 Japan; 2 Department of Bioresource Development, Tokyo University of Agriculture, Funako 1737, Atsugi, Kanagawa 243-0034 Japan; 3 Faculty of Agriculture, Kobe University, 1-1, Rokkodai, Nada, Kobe, Hyogo 657-8501 Japan; 4 Department of Botany, National Museum of Nature and Science, Amakubo 4-1-1, Tsukuba, Ibaraki 305-0005 Japan; 5 Graduate School of Science, Technology and Innovation, Kobe University, 1-1, Rokkodai, Nada, Kobe, Hyogo 657-8501 Japan; 6 Engineering Biology Research Center, Kobe University, 7-1-49, Minatojima Minami Machi, Chuo-ku, Kobe, 650-0047 Japan; 7 Graduate School of Agricultural Science, Kobe University, 1-1, Rokkodai, Nada, Kobe, Hyogo 657-8501 Japan

**Keywords:** White seed, RAD-seq, Genome editing, Lettuce, R2R3-MYB transcription factor

## Abstract

**Key message:**

We identified *LsMybW* as the allele responsible for the shift in color from black to white seeds in wild ancestors of lettuce to modern cultivars.

**Abstract:**

Successfully selected white seeds are a key agronomic trait for lettuce cultivation and breeding; however, the mechanism underlying the shift from black—in its wild ancestor—to white seeds remains uncertain. We aimed to identify the gene/s responsible for white seed trait in lettuce. White seeds accumulated less proanthocyanidins than black seeds, similar to the phenotype observed in *Arabidopsis TT2* mutants. Genetic mapping of a candidate gene was performed with double-digest RAD sequencing using an F_2_ population derived from a cross between “ShinanoPower” (white) and “Escort” (black). The white seed trait was controlled by a single recessive locus (48.055–50.197 Mbp) in linkage group 7. Using five PCR-based markers and numerous cultivars, eight candidate genes were mapped in the locus. Only the *LG7_v8_49.251Mbp_HinfI* marker, employing a single-nucleotide mutation in the stop codon of *Lsat_1_v5_gn_7_35020.1*, was completely linked to seed color phenotype. In addition, the coding region sequences for other candidate genes were identical in the resequence analysis of “ShinanoPower” and “Escort.” Therefore, we proposed *Lsat_1_v5_gn_7_35020.1* as the candidate gene and designated it as *LsMybW* (*Lactuca sativa* Myb White seeds), an ortholog encoding the R2R3-MYB transcription factor in *Arabidopsis*. When we validated the role of *LsMybW* through genome editing, *LsMybW* knockout mutants harboring an early termination codon showed a change in seed color from black to white. Therefore, *LsMybW* was the allele responsible for the shift in seed color. The development of a robust marker for marker-assisted selection and identification of the gene responsible for white seeds have implications for future breeding technology and physiological analysis.

**Supplementary Information:**

The online version contains supplementary material available at 10.1007/s00299-023-03124-4.

## Introduction

**Fig. 1:**
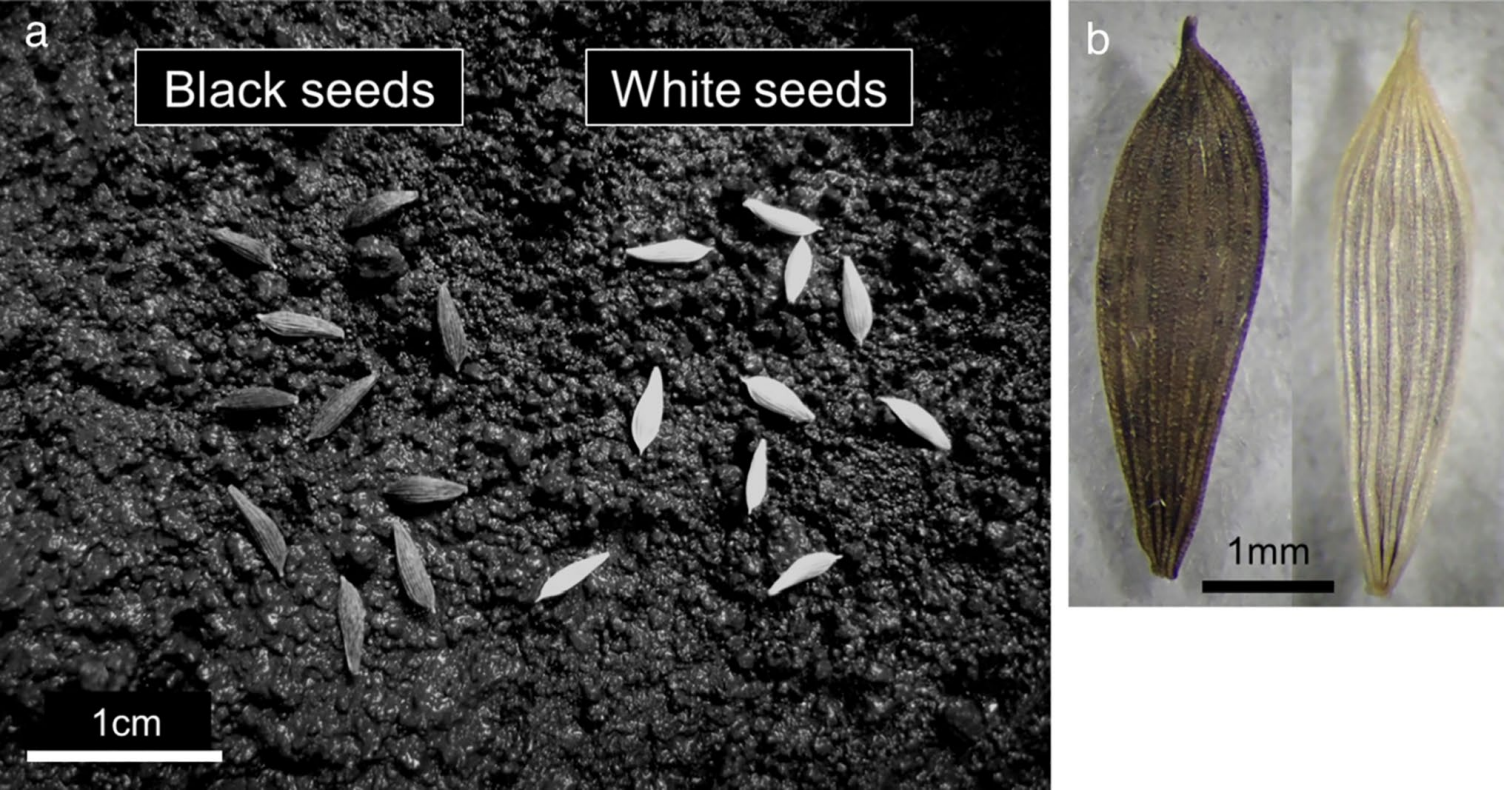
Comparison of black and white seeds of lettuce. **a** Black seeds “Escort” and white seeds “ShinanoPower” shown on the soil surface. **b** Black and white seeds shown on a background of intermediate color

In lettuce (*Lactuca sativa* L.), the major seed colors are black and white. Black-colored seed is the wild-type trait observed in *Lactuca serriola* L., a wild lettuce species distributed worldwide. The white-colored seed trait was possibly discovered during the process of domestication. We infer that individual plants with extremely reduced pigments in the seed pericarp, where the pigments are localized in lettuce, were accidentally discovered (Thompson 1942). Black seeds are difficult to identify on the soil but white seeds could be easily identified on the soil (Fig. 1a, b). Lettuce seeds are small; therefore, the easy visibility that a white seed offers is an advantage in seeding and harvesting. White seeds are desirable for agricultural production. Lettuce seeds are sown near the soil surface as a standard practice (Woolley and Stoller 1978) because the germination of lettuce seeds is promoted by light radiation (Borthwick et al. 1952). Light transmission at 660 and 730 nm induces the germination of black and white seeds, respectively. The transmission spectra of black seeds show transmissions of less than 20% of the incident light, whereas that of white seeds indicate a transmission of more than 50% of the incident light (Widell and Vogelmann 1988). In addition, white seeds are more sensitive to temperature than black seeds, allowing white seeds to germinate with appropriate temperature even in darkness (Borthwick et al. 1952). In maize, it has been reported that the germination rate of light-colored seeds is higher than that of dark-colored seeds under optimum temperature conditions and vice versa in high temperature (Deng et al. 2015). It is considered that darker colored seeds can adapt to poor environments due to the antioxidant capacity of their pigments (Slavin et al. 2009). However, this trait is a rather negative factor in artificially controlled moderate environments, as it leads to, e.g., slower initial imbibition rates during germination (Chachalis and Smith 2000). Therefore, white seeds are believed to be more advantageous and exhibit better agronomic performance and germination than black seeds. The white seed trait is common and is present in well-known cultivars, such as cv “New York.” Several white seed varieties are available in the database; therefore, we believe that the varieties were bred through artificial selection (Table S1). Though the white seed trait is recessive (Thompson 1942; Ryder 1999; Wang et al. 2016), the data of seed lists of the Centre for Genetic Resources, the Netherlands (CGN: https://www.wur.nl/en/Research-Results/Statutory-research-tasks/Centre-for-Genetic-Resources-the-Netherlands-1.htm) and the Germplasm Resources Information Network (GRIN: https://www.ars-grin.gov/) show that the number of white seed cultivars is significantly higher than that of black seed cultivars (Table 1). This fact implies that lettuce breeders around the world intentionally have introduced the trait of white seed into new breeding cultivars. The white seed is an important agricultural trait for lettuce breeders; however, the molecular mechanism underlying the shift from black to white remains incompletely understood (Thompson 1942; Waycott et al. 1999; Kwon et al. 2013). White seed trait is controlled by a recessive single gene (Waycott et al. 1999) located on LG7 (Kwon et al. 2013). We applied the double-digest RAD sequencing (ddRAD-seq) method to analyze the genetic details of the white seed. Further mapping was performed using 84 and 131 cultivars to narrow down the gene, and the only candidate gene identified was validated for involvement in seed color using a knockout mutant through genome editing.

**Table 1:** Number of black seed and white seed cultivars of lettuce germplasm

Plant resource	Total	Black seed	White seed
CGN	1453	512	941
GRIN	2441	1127	1314
CGN + GRIN	3894	1639	2255

## Materials and methods

### Plant material

The crisphead-type lettuce cultivar, “ShinanoPower” was bred by the Nagano Vegetable and Ornamental Crops Experiment Station, and “Escort” was bred by Takii & Co., Ltd. “ShinanoPower” and “Escort” were crossed to produce an F_1_, which was selfed. Approximately 96 F_2_ individuals were investigated for seed color in a greenhouse. The oilseed-type lettuce cultivar “Oilseed” derived from upper Egypt was introduced from CGN; the original strain number is “CGN04769.”

## Analysis of the pigment in seed

White and black seeds were frozen in liquid nitrogen and powdered. The samples were stored at −40 °C until use. The proanthocyanidin content of each seed was compared using vanillin–sulfuric acid assay modified from Sugawara et al. (https://www.naro.affrc.go.jp/project/results/laboratory/karc/2004/konarc04-22.html; accessed February 10, 2020). The freeze-dried powder (100 mg) of each seed was extracted using 1 mL of methanol with shaking. The extracts were mixed with 2 mL 1% (w/v) vanillin/methanol, and 2 mL 25% (v/v) sulfuric acid/methanol was added to the solution and shaken at 30 °C for 15 min. An additional 1 mL of methanol was added to the solution. After centrifugation (3000 rpm/min, 10 min), the absorbance of the supernatant was measured at 500 nm using a Shimadzu UV-2600 UV–Vis spectrometer (Shimadzu Corporation, Japan). To characterize the composition of phenolic compounds, including anthocyanins and other flavonoids, seed powder (100 mg) was extracted with formic acid/H_2_O/methanol (5:10:85, v/v/v). The extracts were filtered with a GL Chromatodisk 13N (0.45 μm pore size, GL Sciences, Inc., Japan) and analyzed using a Shimadzu Prominence HPLC system with a SunShell C18 column [2.6 μm particle material, I.D. 4.6 × 100 mm (ChromaNik Technologies Inc., Japan)] at a flow rate of 0.4 mL/min, detection wavelength of 190–700 nm, and eluent: phosphoric acid/acetonitrile/acetic acid/H_2_O of 3:6:8:83 (v/v/v/v). The injection volume was 1 μL.

## Double-digest RAD sequencing (ddRAD-Seq) and resequencing

Genomic DNA was extracted from the leaves using a NucleoSpin Plant II Extract Kit (Machery-Nagel, Duren, Germany). ddRAD-seq and resequencing were performed as described by Seki et al. (2020) and Seki (2021). The ddRAD-seq libraries were sequenced using the Illumina Hiseq4000 platform. Paired-end sequencing reads (100 bp × 2) were analyzed for ddRAD-seq tag extraction, counting, and linkage map construction using RAD-R scripts (Seki 2021). The linkage map was graphically visualized using MapChart (Voorrips 2002). Resequencing libraries were sequenced on the HiSeqX platform. DNA samples from the two parental lines were used to construct paired-end sequencing libraries (150 bp × 2) and were subjected to whole-genome sequencing. Raw sequence data (fastq) for the present RAD-seq and resequence analysis are available in the DNA Data Bank of Japan (DDBJ) Sequence Read Archive (SRA: http://ddbj.nig.ac.jp/dra/index_e.html) under accession number DRA010289.

## Resequence analysis of parent genomes and sequencing of a candidate locus

Resequencing reads from the two parental cultivars were mapped onto the lettuce reference genome [version 8 from the crisphead cultivar “Salinas”; (https://genomevolution.org/coge/GenomeInfo.pl?gid=28333)] (Reyes-Chin-Wo et al. 2017) using the BWA software (Li and Durbin 2009). A detailed script is provided in Resequence_mapping_script_BWA_mem.txt (https://github.com/KousukeSEKI/RAD-seq_scripts). The sorted BAM files were visualized using the IGV software (Robinson et al. 2011).

## Genotyping using publicly available genome resequencing data

The publicly available *L. sativa* genome sequencing data were obtained from the NCBI SRA as shown in Table S2. FASTQ files were imported into the CLC Genomics Workbench (QIAGEN, USA) for subsequent analysis. The trim sequence tool in the suite was used to filter out low-quality bases (< Q30), and only reads that showed a quality score of ≥ 30 were retained. Filtered sequence reads were mapped onto the *L. sativa* v8.0 genome (https://phytozome.jgi.doe.gov/pz/portal.html#!info?alias=Org_Lsativa_er) using the Map Reads Reference tool, and local realignment was performed using the Local Realignment tool. Based on the mapping results, the genomic polymorphisms associated with the white seed trait were identified.

## Designing PCR-based markers and their amplification

Polymorphisms near the locus at 48–50 Mbp in LG7, including insertions, deletions, and single-nucleotide polymorphisms (SNPs), were evaluated as potential markers. The primer names were formatted as (linkage group) _ (genome version) _ (genome position) _ (restriction enzyme, in case of CAPS). Primers for locus amplification were designed using Primer3 (http://bioinfo.ut.ee/primer3-0.4.0/), and KOD FX (TOYOBO, Japan) was used for amplification. PCR was performed using 0.5 μL of DNA template, 0.4 μL of each primer (50 μM), 2 μL of dNTP (2 mM), 5 μL of 2 × PCR Buffer, 0.2 μL of KOD FX (1 U/μL), and distilled water (dH_2_O) to a final reaction volume of 10 μL. The PCR conditions were as follows: 94 °C for 5 min, 30 cycles of 94 °C for 30 s and 62 °C for 30 s, followed by one cycle at 72 °C for 4 min. After amplification, electrophoresis was performed using 9 μL of the PCR products on a 2% agarose gel (Takara Bio, Japan) at 100 V. In case of CAPS, the PCR products were digested at 37 °C for 1 h in 20 µL total volume with 5 − 15 units of the appropriate restriction enzyme before electrophoresis.

## Genome editing

The 20-nt gRNAs specific for the target gene, *LsMybW* were designed for the first exon (Fig. S1 and Table S3). Primers with the *Bbs*I restriction site were annealed and ligated into the entry vector, 1480_MluI-1433_pUC19_AtU6oligo (Shimatani et al. 2017). The core partial fragments were excised from the entry vector using *I-Sce*I and subcloned into the T-DNA region of the destination vector, 1432_pZD_OsU3gYSA_HolgerCas9_NPTII (Shimatani et al. 2017). All vectors were constructed through standard cloning and verified through sequencing. Lettuce “Oil seed” was transformed with Agrobacterium harboring the destination vector. Regenerated plants were selected using kanamycin, and genome integration of T-DNA was confirmed through PCR using NPTII. To determine the genome-editing ability of *LsMybW*, the target sequence was amplified from gDNA through PCR and cloned into the standard sequencing vector, pSKII^−^. Eight clones of each T_0_ strain were used in this study.

## Results

### Analysis of pigment accumulation in seeds

The absorbances of the supernatants extracted from black and white seeds at 500 nm were 0.061 (SE = 0.015) and 0.038 (SE = 0.008), respectively. The proanthocyanidin content in the white seed was 0.62 times lower than that in the black seeds. Some phenylpropanoids such as chlorogenic acid were found in both seed colors; however, common anthocyanins and other flavonoids were not detected in the HPLC analysis (Figs. S2, S3). White seeds appear to have reduced function with regard to the accumulation of proanthocyanidins.

## Inheritance of seed color

An F_2_ population was derived from an initial cross between “ShinanoPower” (white seed) and “Escort” (black seed) to elucidate the inheritance of the white seed trait. The F_1_ plants produced black seeds. Among the 96 F_2_ individuals, 74 possessed black seeds and 22 possessed white seeds, fitting 3:1 ratio with the Chi-square test (*p* = 0.64). These results suggest that the white-seed trait is determined by a single recessive locus.

## Double-digest RAD sequencing analysis of the F_2_ population and linkage map development

The ddRAD-seq analysis was used to genotype the F_2_ population for genetic mapping of the locus for the white seed trait derived from “ShinanoPower.” The genomic DNA polymorphisms between the two parental lines, “ShinanoPower” and “Escort,” were assessed through ddRAD-seq analysis using PacI and NlaIII restriction enzymes (Seki et al. 2020). Illumina HiSeq sequencing of the ddRAD-seq libraries produced 9,281,482 and 8,107,399 single reads (100 bp) for “ShinanoPower” and “Escort” plants, respectively. RAD-tags were extracted from the sequence reads of individual samples. In total, 346,396 and 302,738 RAD-tags with more than 2 read counts were obtained for the “ShinanoPower” and “Escort” samples, respectively. Comparing the RAD-tags of the 2 parental lines, 135,129 and 91,471 unique tags were identified as either “ShinanoPower”- or “Escort”- specific tags, respectively, whereas 211,267 RAD tags appeared in both samples. Read mapping was performed with the unique RAD tags of each parent against the reference lettuce genome sequence. A total of 2871 pairs of RAD tags (designated as biallelic tags) harboring SNPs or InDels from the two parental lines were described, and these biallelic tags were employed as co-dominant markers for further genetic mapping. By summarizing co-segregated biallelic tag loci, 1,038 loci were regarded as the co-dominant markers. The genotypes of these 1038 biallelic tagged loci were also determined through the ddRAD-seq analysis of 96 individuals in the F_2_ population. Genotypes of the biallelic tag loci in the 96 F_2_ individuals were determined based on the presence or absence of each allelic tag. After excluding loci with missing data, genotyping data from 856 biallelic tag loci of 96 F_2_ individuals were used for linkage map construction (Fig. S4 and Table 2). Based on the grouping analysis, the marker loci were distributed into nine linkage groups. Ordering the marker loci in each linkage group resulted in a linkage map comprising 988.8 cM (Fig. S4). Summary statistics for the linkage maps are presented in Table 2. Marker density ranged from 0.5 cM per marker (LG7) to 4.2 cM per marker (LG9). The number of markers ranged from 22 (LG9) to 177 (LG7).

**Table 2:** Summary of integrated lettuce linkage groups

Linkage groups	Total mapped tags	Common tags		Escort unique tags		ShinanoPower unique tags		Linkage construct marker		Average interval between markers
		No. RAD-tags	(%)	No. RAD-tags	(%)	No. RAD-tags	(%)	No. biallelic tags	Map length (cM)	(cM)
LG1	37,235	15,284	41.0	8481	22.8	13,470	36.2	138	105.5	0.8
LG2	39,121	18,603	47.6	8830	22.6	11,688	29.9	67	43.0	0.6
LG3	51,405	25,399	49.4	10,965	21.3	15,041	29.3	55	152.4	2.8
LG4	73,106	36,016	49.3	14,848	20.3	22,242	30.4	113	141.0	1.2
LG5	64,803	31,929	49.3	13,564	20.9	19,310	29.8	101	177.7	1.8
LG6	36,432	18,005	49.4	7143	19.6	11,284	31.0	76	87.1	1.1
LG7	36,744	17,301	47.1	7844	21.3	11,599	31.6	177	88.6	0.5
LG8	60,517	29,382	48.6	12,273	20.3	18,862	31.2	107	100.5	0.9
LG9	38,504	19,348	50.2	7523	19.5	11,633	30.2	22	93.0	4.2
Total	437,867	211,267	48.2	91,471	20.9	135,129	30.9	856	988.8	1.2

## Fine mapping of the white seed locus and candidate gene analysis

**Fig. 2:**
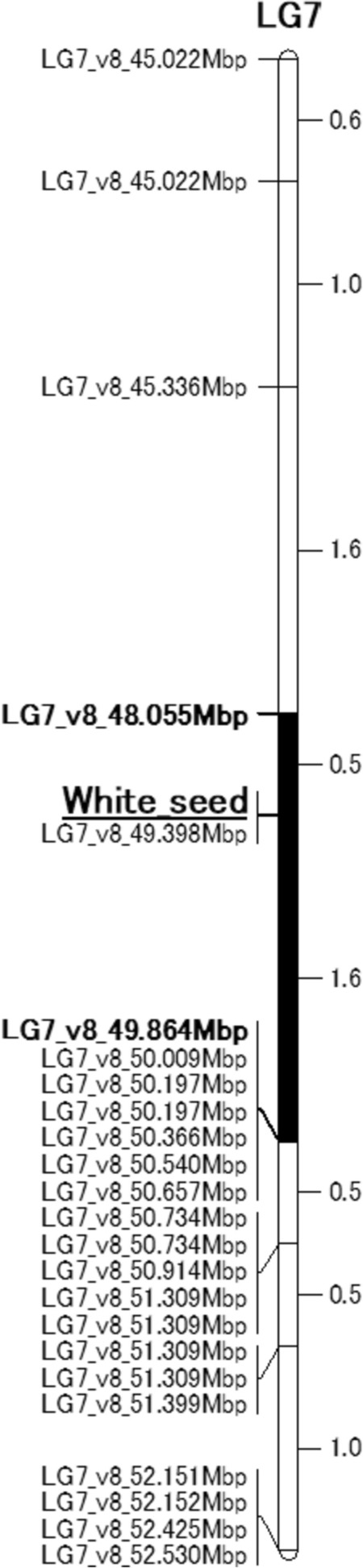
The mapped location of the white seed locus on LG7. Genetic distances (cM) are shown between the markers. “White seed” indicates the position of the responsible gene for the trait of white seed. The black bar indicates the white seed locus

**Fig. 3:**
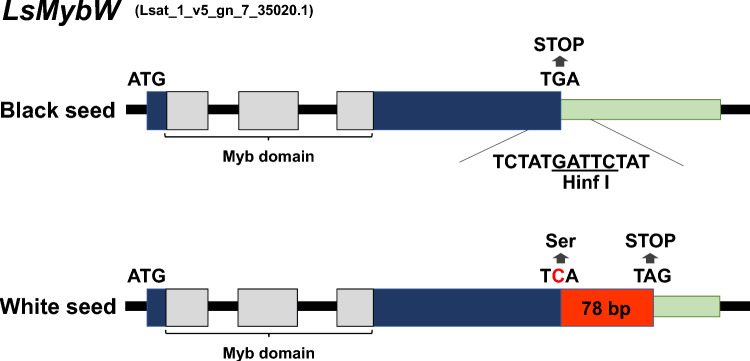
Comparison of *Lsat_1_v5_gn_7_35020.1* between black and white seeds. This white seed allele encodes an additional 78 bp at the 3′ end that are not present in black seed allele. The black seed allele sequence contains a Hinf I restriction site in the stop codon

**Fig. 4:**
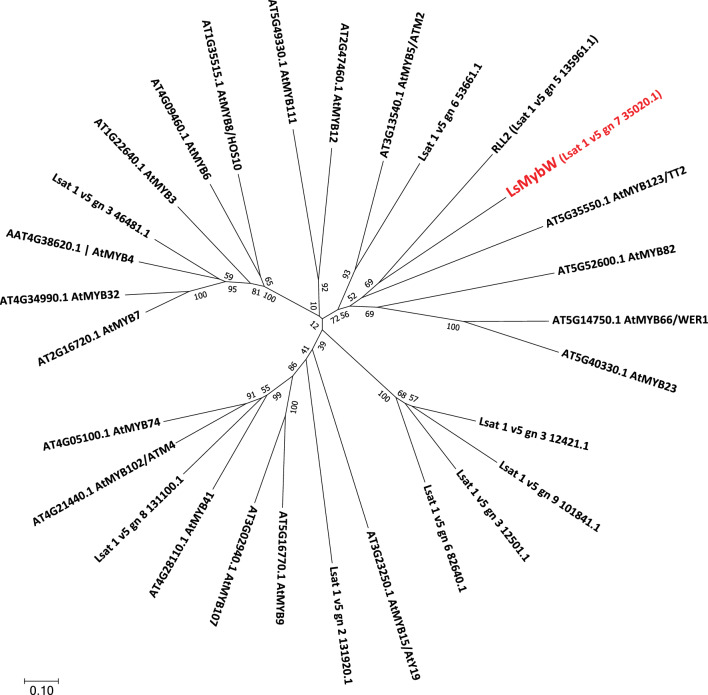
Phylogenetic analysis of the MYB domain-containing proteins in *L. sativa*. The evolutionary tree was built with deduced amino acid sequence from orthologs encoding MYB transcription factor in lettuce and *Arabidopsis*. The red font indicates the candidate gene controlling seed color in lettuce

For genetic mapping of the locus for white seed trait, ddRAD-seq analysis was conducted for constructing a linkage map by RAD-R scripts using F_2_ population. A single locus tightly linked to the white-seed trait was located in LG7 and flanked by two markers (*LG7_v8_48.055 Mbp* and *LG7_v8_49.864 Mbp*) based on the genotypes of the biallelic RAD-tags. The marker designated as *LG7_v8_49.398 Mbp* exhibited complete co-segregation with the white seed trait within the F_2_ population (Fig. 2). Moreover, fine mapping of the target locus was performed using 5 markers (Table S4) and 84 cultivars (Table S5). Forty-five cultivars had white seeds and the rest had black seeds. Only the *LG7_v8_49.251Mbp_HinfI* marker was associated with the white seed phenotype (Tables 3, S5). Based on the marker data, it was predicted that the responsible gene was located between 49.173 and 49.326 Mbp in LG7. Eight open reading frames (ORFs) were positioned in this region according to the annotated reference genome sequence of *L. sativa* V8 (Table 4). The sequences of these eight ORFs were compared between “ShinanoPower” and “Escort.” There were no small InDels or nonsynonymous substitutions in these seven ORFs between the two parental lines; however, a single-nucleotide mutation in a stop codon was found in ORF 7, which is referred to as *Lsat_1_v5_gn_7_35020.1*. The allele of the white seed cultivar encoded an additional 78 bp at the 3′ end that was not present in the black seed allele (Figs. 3, S5). Based on the analysis using publicly available resequencing data from 131 cultivars, only the stop codon polymorphism showed a complete correlation with seed color in 21 polymorphisms in the genomic region from 49.173 to 49.326 Mbp (Tables 5, S2, S6). Phylogenetic analysis revealed that *Lsat_1_v5_gn_7_35020.1* is closely related to the *TRANSPARENT TESTA 2* (*TT2*) gene encoding the R2R3-MYB transcription factor (Fig. 4), which is involved in the regulation of seed color in *Arabidopsis thaliana* (Nesi et al. 2001); we named it as *LsMybW* (*Lactuca sativa* Myb White seeds). R2R3-MYB forms the MYB-bHLH-WDR (MBW) ternary protein complex together with bHLH-type transcriptional regulators and the WD repeat protein (Lepiniec et al. 2006). The MBW complex regulates the transcription of gene subsets related to anthocyanin and proanthocyanidin synthesis, thereby modulating the pool size of these metabolites. In addition, the paralog with the greatest similarity, *Lsat_1_v5_gn_5_135961.1*, was related to the regulation of anthocyanin biosynthesis in the leaf of red leaf cultivars and was named as Red Lettuce Leaf 2 (RLL2) (Su et al. 2020).

**Table 3:** Seed color and genotype of multiple markers in 84 lettuce cultivars

Seed color	Genotype	Expected genotype	Marker name				
			LG7_v8_48.637Mbp	LG7_v8_49.173Mbp_EcoRI	LG7_v8_49.251Mbp_HinfI	LG7_v8_49.326Mbp	LG7_v8_49.798Mbp
White	ShinanoPower (White seed coat)	45	33	34	45	40	41
	Escort (Black seed coat)	0	12	11	0	5	4
Black	ShinanoPower (White seed coat)	0	10	7	0	8	4
	Escort (Black seed coat)	39	29	32	39	31	35

**Table 4:** Candidate genes in the genomic region between 49.173 and 49.326 Mbp in LG7

ORF	Gene model name	Position of reference genome sequence		Putative function	Sequencing
		Start	End		
ORF1	Lsat_1_v5_gn_7_35140.1	49,203,264	49,205,806	Subtilase family protein, putative	Identical
ORF2	Lsat_1_v5_gn_7_35121.1	49,225,195	49,229,412	FRIGIDA interacting protein, putative	Identical
ORF3	Lsat_1_v5_gn_7_35101.1	49,231,640	49,234,256	Acyl-CoA N-acyltransferases (NAT) superfamily protein, putative	Identical
ORF4	Lsat_1_v5_gn_7_35080.1	49,234,299	49,235,990	Mitochondrial editing factor, putative	Identical
ORF5	Lsat_1_v5_gn_7_35060.1	49,239,808	49,242,933	RNI-like superfamily protein, putative	Identical
ORF6	Lsat_1_v5_gn_7_35041.1	49,250,652	49,252,131	TATA-binding related factor (TRF) of subunit 20 of mediator complex, putative	Identical
ORF7	Lsat_1_v5_gn_7_35020.1	49,251,738	49,252,982	R2R3-MYB transcriptional factor, putative	A single-nucleotide mutation in stop codon
ORF8	Lsat_1_v5_gn_7_34980.1	49,324,772	49,325,209	ELF4-like, putative	Identical

**Table 5:** Seed color and genotype of multiple markers in 131 lettuce cultivars

Seed color	Genotype	Expected genotype	Marker name or genomic position in LG7 (bp)										
			LG7_v8_49.173Mbp EcoRI	49,216,366	49,221,539	49,240,810	49,252,085	49,253,112	49,279,929	49,286,547	49,299,431	49,325,952	49,325,999
White	ShinanoPower (white seed coat)	81	68	58	55	58	81	60	59	80	80	76	75
	Escort (black seed coat)	0	13	23	23	21	0	21	22	1	1	5	6
	Other or no detected^a^	0	0	0	3	2	0	0	0	0	0	0	0
Black	ShinanoPower (white seed coat)	0	27	20	24	18	0	1	1	30	30	10	10
	Escort (black seed coat)	50	23	30	25	27	50	49	49	20	20	40	40
	Other or no detected^a^	0	0	0	1	5	0	0	0	0	0	0	0

Seed color	Genotype	Expected genotype	Marker name or genomic position in LG7 (bp)									
			49,326,246	49,326,534	49,326,551	49,326,571	49,326,672	49,326,707	49,326,711	49,326,749	49,326,756	LG7_v8_49.326Mbp
White	ShinanoPower (white seed coat)	81	75	65	57	71	76	76	76	76	76	76
	Escort (black seed coat)	0	5	5	5	5	5	5	5	5	5	5
	Other or no detected^a^	0	1	11	19	5	0	0	0	0	0	0
Black	ShinanoPower (white seed coat)	0	30	26	26	29	30	30	30	30	30	30
	Escort (black seed coat)	50	20	20	20	20	20	20	20	20	20	20
	Other or no detected^a^	0	0	4	4	1	0	0	0	0	0	0

^a^See Table S6 for detailed genotypes

## Validation of *LsMybW* function through CRSPR/Cas9-based genome editing

**Fig. 5:**
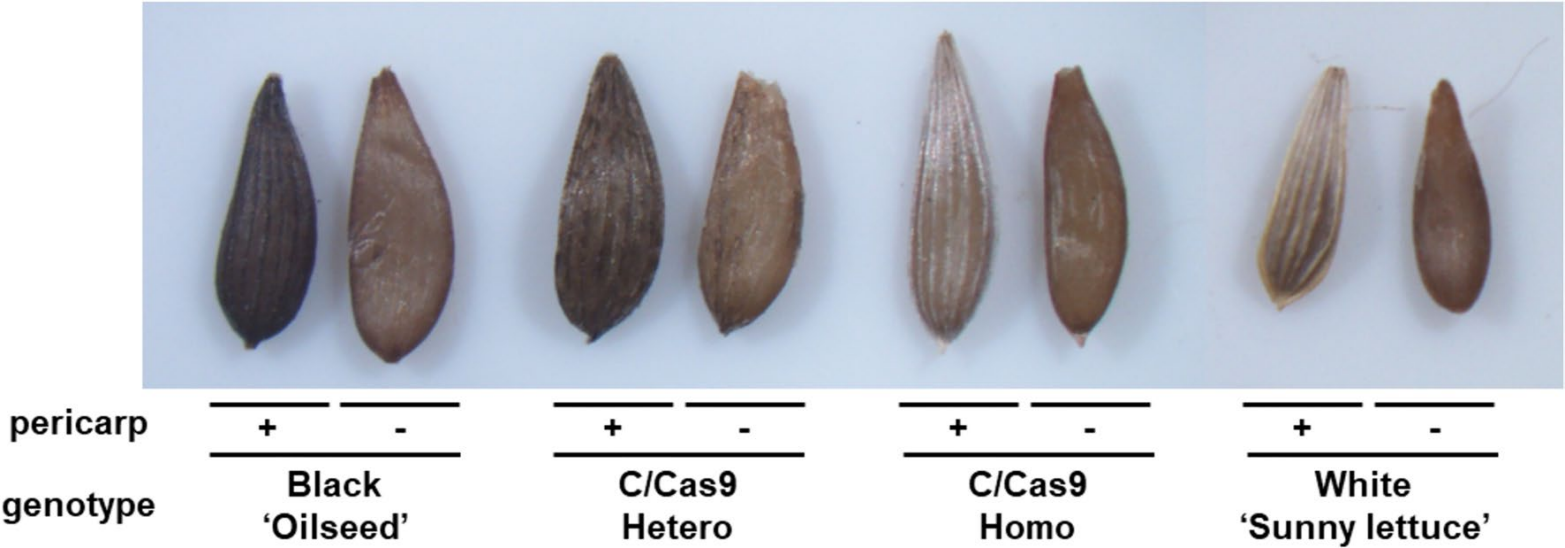
Characterization of seed color of genome-edited lettuce. The outermost appearance was determined alternatively as black or white. Pericarp was removed from lettuce achene (−) or not ( +). “Oilseeds” is a black seed cultivar, and T_1_ seed is from genome-edited plants (C/Cas9), while “Sunny lettuce” is a white seed cultivar. Heterozygous (Hetero) or homozygous mutant (Homo) was determined based on the CRISPR/Cas9-induced mutation. The seed color described here is derived from the parental T_0_ trait

To obtain knockout strains with a loss-of-function mutation in the *LsMybW* gene, gRNA was designed at the first exon to generate the early stop codon. CRISPR/Cas9 vector was introduced via Agrobacterium into the lettuce, “Oilseed,” which normally produces black seeds. The transformed plants were selected for both antibiotic resistance and PCR-positive results for foreign genes. Nine acclimated T_0_ individuals were used for the analysis of the target sequence. The resulting sequence variations of the eight clones allowed us to predict the genotype of the strains: monoallelic or biallelic, heterozygous or homozygous, in-frame or out-of-frame. Six strains produced white seeds in the T_0_ phenotype (Table 6). Among these, three strains (4-1, 15-1, 19-2) could be biallelic homozygous mutants with a single-base insertion. The other three strains (5-1, 30-4, 22-2) that produced white seeds were biallelic heterozygous mutants with insertions of one or two bases. All edits generated early stop codons and 13 (MGRSPCLFKDWSE*) or 12 (MGRSPCLVQRLV*) short peptides. The three remaining strains (15-2, 25-4, 19-1) had black seeds, despite genome editing. This could be attributed to the presence of biallelic heterozygous mutants, including in-frame editing with a three-base deletion. These results confirmed that *LsMybW* controls the dominant traits of seed color. The inner parts when observed without the pericarp were brown in all genotypes (Fig. 5), suggesting that *LsMybW* is involved only in achene color.

**Table 6:**
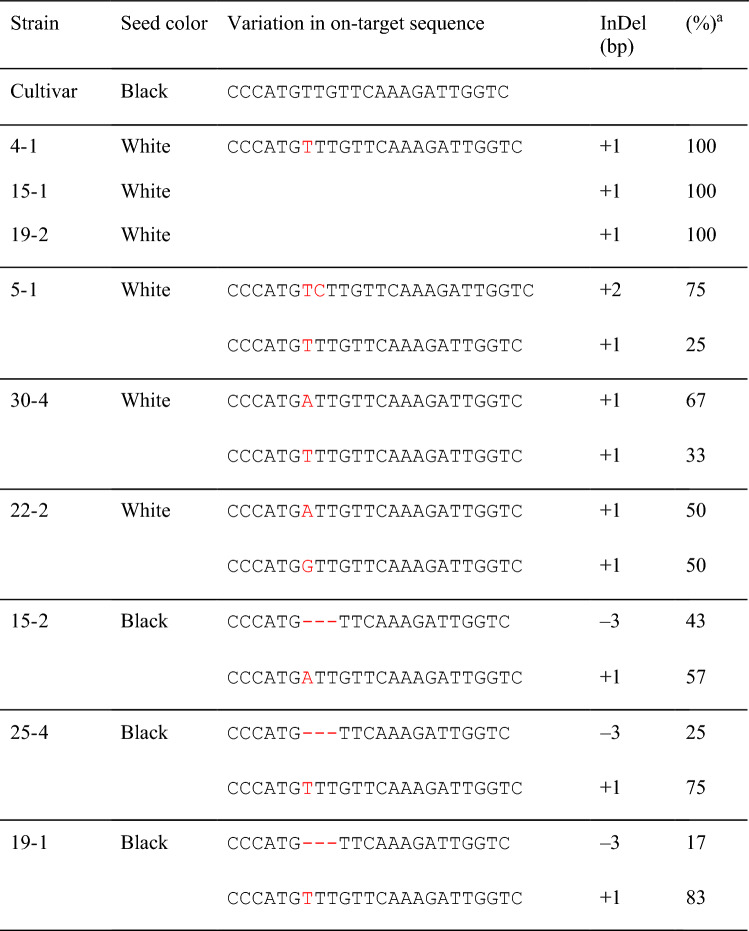
Seed color and sequence variation of genome-edited *LsMybW* gene in T_0_ plants

Red characters indicate InDel sequences by genome-editing

^a^Percent of sequenced clones to total (*n* = 7–8)

## Discussion

In this study, we succeeded in the genetic mapping and identification of the genes responsible for white seeds in lettuce. According to genetic mapping using ddRAD-seq and PCR-based markers, the locus of white seeds was located between 49.173 and 49.326 Mbp in LG7 (Fig. 2 and Table 3). Eight predicted genes were identified in this region (Table 4). Resequence analyses in “ShinanoPower” (white seed) and “Escort” (black seed) revealed that the candidate genes of the coding sequences were identical except for *LsMybW*, gene model name *Lsat_1_v5_gn_7_35020.1* (Table 4). *LsMybW* has a single-nucleotide mutation in the stop codon of cultivars with white seeds, and the *LG7_v8_49.251Mbp_HinfI* marker, which employs this mutation, was completely linked to the white seed phenotype (Fig. 3 and Table 3). Analysis of the publicly available resequencing data revealed a full correlation between stop codon polymorphisms and seed color (Table S2). In addition, this marker position overlapped that of a previously reported locus for white seed color (Kwon et al. 2013; Simko et al. 2013). Pigment accumulation in plants is controlled by two gene subsets: early and late biosynthetic genes (LBGs) (Kubasek et al. 1992; Quattrocchio et al. 1993; Nesi et al. 2000). The transcription factor complex, consisting of *AtTT2*, *AtTT8*, and *AtTTG1*, controls the expression of LBGs (Gonzalez et al. 2008, 2016), and *AtTT2* mutants have an altered seed color (yellow) due to the absence of proanthocyanidin production in *Arabidopsis* (Shirley et al. 1995; Nesi et al. 2001). *AtTT2* controls the expression of *BAN* (anthocyanidin reductase gene), which is involved in the divergence of proanthocyanidins and anthocyanins during flavonoid biosynthesis (Debeaujon et al. 2003). Therefore, as it occurs in *Arabidopsis*, proanthocyanidins may be responsible for seed color in lettuce rather than anthocyanins (Fig. S2). *LsMybW* shared 28% of their identity with *AtTT2* and highly conserved a DNA-binding domain-containing R2 and R3 repeats, consisting of ‘-W-(X19)-W-(X19)-W-’ and ‘-F/I-(X18)-W-(X18)-W-’, respectively. Therefore, *LsMybW* is a biologically plausible candidate gene. Genome editing of the target *LsMybW* showed that knockout mutants harboring an early termination codon produced white seeds (Table 6, Fig. 5). We infer that the orthologous proteins in lettuce probably have a conserved function; the mutation of the stop codon of *LsMybW* in white seeds causes a significant conformational change and interferes in complex formation and other interactions. Dominant-negative effects of MYB due to deletions in the C-terminal end have been reported in *Arabidopsis* (Velten et al. 2010). Within the amino acid sequence of an additional 26 residues at the C-terminus, we could not find any typical repressor motifs for MYB, such as the ERF-associated amphiphilic repression (LxLxL or DLNxxP), Sensitive to ABA and Drought 2 protein interact motif (GY/FDFLGL), or TLLLFR (Wu et al. 2022). Analyzing additional sequences is worth exploring, including the possibility of identifying novel inhibitory motifs. The white seeds exhibited reduced accumulation of proanthocyanidins compared to the black seeds based on the vanillin–sulfuric acid assay, which is in agreement with the conclusion that the white seed cultivars have a reduced-function mutation in *LsMybW* that controls the expression of LBGs in lettuce seeds. In conclusion, *LsMybW* is the allele responsible for the shift in seed color from black to white.

During the artificial crossing of lettuce flowers, the breeder must remove the maternal parent pollen from the flower. However, it is impossible to completely remove pollen from lettuce flowers; therefore, seeds of both the selfed progeny and the F_1_ hybrid are produced unintentionally owing to a compound autogamous floral structure (Simko et al. 2011). Therefore, breeders would like to utilize the inheritance patterns of easily recognizable traits to distinguish F_1_ hybrids from selfed plants in the following progeny. Following a cross between white seed pure line (♀) and black seed pure line (♂), selfed progenies produce the next generation of white seed and F_1_ hybrids produce the F_2_ generation of black seed because of the dominant trait (Thompson 1942; Ryder 1999). Therefore, seed color can be effectively used to distinguish between selfed and hybrid plants (Thompson 1942). The marker used to distinguish between F_1_ hybrids and selfed plants in populations derived from the white seed × black seed cross could also contribute to validating the phenotype of the F_1_ generation. The verification of inconspicuous traits that require bioassays, such as disease resistance, has been difficult in the F_1_ generation. Male sterility makes it possible to produce only F_1_ hybrid seeds (Hayashi et al. 2011; Seki 2022); however, it has not been widely used because of limited cross combinations. The inheritable characteristics of the F_1_ generation can be examined using a bioassay with only the seeds of the hetero genotype. From the F_2_ generation onward, it was possible to intentionally select the seed color using the marker. These approaches are valuable for the development of breeding methods that accelerate the development of lettuce cultivars. Therefore, *LG7_v8_49.251Mbp_HinfI* marker-targeted *LsMybW* could be used to distinguish almost all white seeds of lettuce worldwide and could be applied to significantly enhance lettuce breeding programs (Tables S1, S2, S4).

Lettuce was first domesticated near the Caucasus after the loss of seed-shattering by spontaneous mutation (Wei et al. 2021). Therefore, cultivated lettuce has a non-seed-shattering characteristic owing to the same *qSHT* locus. Because the domestication time is estimated to be around 4000 BC, the change in seed color is even later. Considering that lettuce was depicted on wall paintings of Egyptian tombs around 2500 BC as one of the major vegetable crops (De Vries 1997), it is reasonable to assume that the change in seed color occurred before the global spread of lettuce seeds (Tables S2, S5). The discovery of white seeds, which have been used as an important agronomic trait for thousands of years, was indeed a great achievement.

## Conclusion

The development of a robust marker for marker-assisted selection and identification of the gene responsible for white seeds has implications for lettuce breeding and agricultural aspects regarding seed color. This study not only identified a gene responsible for the white seed phenotype, but also revealed an important gene regulating a key agronomic trait for lettuce cultivation and breeding. These findings could be useful for future lettuce breeding endeavors.

### Supplementary Information

Below is the link to the electronic supplementary material.

Supplementary file1 (DOCX 532 KB)

Supplementary file2 (XLSX 48 KB)

## Data Availability

Raw sequence data (FASTQ) for the ddRAD-seq dataset were deposited in the DNA Data Bank of Japan (DDBJ) Sequence Read Archive (http://ddbj.nig.ac.jp/dra/index_e.html) under the accession number DRA013652.
