# Supplementary material for: LsMybW-encoding R2R3-MYB transcription factor is responsible for a shift from black to white in lettuce seed
Source: Plant Cell Rep. 2024 Jan 11;43(2):35. doi: 10.1007/s00299-023-03124-4 (PMC10781863; doi:10.1007/s00299-023-03124-4)
Supplement: Supplementary file 1 — Supplementary file1 (DOCX 532 KB) [file 299_2023_3124_MOESM1_ESM.docx]

Supplementary Material

*LsMybW*-encoding R2R3-MYB transcription factor is responsible for a shift from black to white in lettuce seed

Kousuke Seki, Kenji Komatsu, Kanami Yamaguchi, Yoshinori Murai, Keiji Nishida, Ryohei Koyama, and Yuichi Uno*

Fig. S1. *LsMybW* nucleotide sequence and gRNA design. The 50 bp of the first exon of *LsMybW* is shown. The red arrow indicates the PAM sequence and the green arrow indicates gRNA. Indels other than multiples of three cause frameshifts and the appearance of an early termination codon.

White seed (520 nm)

Black seed (340 nm)

Chlorogenic acid

White seed (340 nm)

Chlorogenic acid

Fig. S2. HPLC chromatograms of black and white seed extracts. Detection of 520 nm was for common anthocyanins, and detection of 340 nm was for other flavonoids and phenylpropanoids. Injection peaks: 2.4-2.8 min, chlorogenic acid: 5.6 min, and other phenylpropanoids: 4.0, 4.3, 7.2, 28.3, 38.5 and 47.6 min.

Fig. S3. UV-vis spectrum of chlorogenic acid (5.6 min) detected from lettuce seed in HPLC survey of this study. The spectra of the other phenylpropanoids were similar.

Fig. S4. Schematic of the consensus map for an F_2_ population derived from a cross between "ShinanoPower" and "Escort". The number on the left indicates the distance in cM and the characters on the right indicate the marker name. Horizontal lines across chromosomes indicate the positions of the loci on each chromosome.

Fig. S4. continued

Fig. S4. continued

Fig. S5. Comparison of *LsMybW* nucleic acid sequences of black and white seeds.
